# The nanomolar sensing of nicotinamide adenine dinucleotide in human plasma using a cycling assay in albumin modified simulated body fluids

**DOI:** 10.1038/s41598-018-34350-6

**Published:** 2018-10-31

**Authors:** Philipp Brunnbauer, Annekatrin Leder, Can Kamali, Kaan Kamali, Eriselda Keshi, Katrin Splith, Simon Wabitsch, Philipp Haber, Georgi Atanasov, Linda Feldbrügge, Igor M. Sauer, Johann Pratschke, Moritz Schmelzle, Felix Krenzien

**Affiliations:** 1 Experimental Surgery and Regenerative Medicine, Department of Surgery, Campus Charité Mitte and Campus Virchow-Klinikum, Charité - Universitätsmedizin Berlin, Berlin, 13353 Germany; 2 Berlin Institute of Health (BIH), Berlin, 10178 Germany

## Abstract

Nicotinamide adenine dinucleotide (NAD), a prominent member of the pyridine nucleotide family, plays a pivotal role in cell-oxidation protection, DNA repair, cell signalling and central metabolic pathways, such as beta oxidation, glycolysis and the citric acid cycle. In particular, extracellular NAD^+^ has recently been demonstrated to moderate pathogenesis of multiple systemic diseases as well as aging. Herein we present an assaying method, that serves to quantify extracellular NAD^+^ in human heparinised plasma and exhibits a sensitivity ranging from the low micromolar into the low nanomolar domain. The assay achieves the quantification of extracellular NAD^+^ by means of a two-step enzymatic cycling reaction, based on alcohol dehydrogenase. An albumin modified revised simulated body fluid was employed as standard matrix in order to optimise enzymatic activity and enhance the linear behaviour and sensitivity of the method. In addition, we evaluated assay linearity, reproducibility and confirmed long-term storage stability of extracellular NAD^+^ in frozen human heparinised plasma. In summary, our findings pose a novel standardised method suitable for high throughput screenings of extracellular NAD^+^ levels in human heparinised plasma, paving the way for new clinical discovery studies.

## Introduction

Nicotinamide adenine dinucleotide (NAD) is a pyridine dinucleotide omnipresent in all living cells either in oxidised (NAD^+^), or reduced (NADH) form, whose ratio dictates the intracellular redox status and thus stipulates the overall cellular metabolic state^1,2^. Extracellular NAD^+^(eNAD^+^) was shown to exhibit important secondary messenger properties and acts to induce intracellular calcium release, thereby mediating lymphocyte chemotaxis^3^. Interestingly, eNAD^+^ is known to be the result of either lytic release from injured tissue or non-lytic release mechanisms through pore forming proteins like connexin 43 (Cx43) hemichannels and is thus hypothesised to mediate immune response and organ function by means of paracrine signalling^4–6^.

Therapeutic applications of eNAD^+^ have been subject to extensive testing in murine models, with striking findings demonstrating anti-aging, regenerative and highly immunomodulatory traits^7,8^. For instance, Tullius *et al*.^9^, discovered that the systemic administration of *β*-NAD^+^ did not only block autoimmune encephalomyelitis induced paraplegia, but reversed disease progression through remyelination and neuroregeneration^9^. Furthermore, eNAD^+^ induced regulatory T cell differentiation, promoting allograft survival in a murine skin transplantation model with implications for concepts of alloimmunity and inflammatory diseases^10^. However, a significant lack of medical translation towards the study of eNAD^+^ in human disease is apparent.

In fact, the measurement of eNAD^+^ in different bodily compartments is challenging due to vast concentration variations. In human erythrocytes, intracellular NAD^+^ (iNAD^+^) was determined to be in the range of 10–40 M, whilst eNAD^+^ in pig plasma was found at a fraction of this concentrations, namely 240–290 nM^11–13^. Therefore, analytical tests have to be inherently robust and span vastly different concentration ranges. High performance liquid chromatography (HPLC) NAD^+^ analysis methods usually require any and all samples to be internally spiked with NAD^+^ and have struggled to quantify NAD due to signal masking and ionisation suppression, especially when followed by ultraviolet-visible (HPLC-UV) spectroscopic quantification^14^. However, HPLC methods followed by mass spectrometry (HPLC-MS), and especially tandem mass spectrometry (HPLC-MS/MS), have managed to achieve remarkable sensitivity and specificity for iNAD^+^ thanks to their ability to separate the respective metabolites through elution with highly specialised columns and successively combining the relaxation time of specific metabolites with their mass spectrum^15^. Generally, the entirety of the apparatus, namely the columns, the mass or UV spectrometer as well as the actual HPLC machine, required for HPLC analyses tends to come with a hefty price tag, rendering it prohibitively expensive for the general scientific community. Typically, each singular measurement of LC-based assays consumes significant amounts of time, from ten minutes up to one hour, making their application to high throughput screenings impractical.

A promising concept enabling the measurement of minuscule analyte concentrations is known as enzymatic cycling, whereby a reactant recycling of the analyte takes place and is used to overproportionately amplify a redox indicator dye mediated signal, without the need for additional purification or concentration steps. First introduced by Warburg *et al*.^16^, the analysis of pyridine nucleotides was pioneered through an enzymatic cycling reaction^17–19^. In an effort to refine this method, Rhodes *et al*.^20^, used alcohol dehydrogenase for the cycling reaction and quantification of pyridine nucleotides in cox orange apples^20^. Although this method relied on the fluorimetric measurement of the highly red fluorescing resorufin, the reduced form of resazurin, the signal might be masked by the autofluorescence of plasma. In later years, ADH gained popularity and was subsequently adopted as the enzyme of choice, as it is present in all living organisms^21–23^. Nonetheless, the majority of these studies have been conducted to investigate iNAD^+^, and have not been validated for the study of eNAD^+^ in human plasma. Consequently, we established a method to meet the challenges of measuring eNAD^+^ in human plasma based upon known protocols^13,20,24^. More precisely, a heat based dichotomous pH extraction procedure was implemented to extract purified versions of NAD^+^ or NADH, subsequently quantified by a colorimetric two-step enzymatic cycling assay^18,25^. The method’s sensitivity ranged from the low micromolar into the low nanomolar domain, using an albumin adjusted revised simulated body fluid (r-SBFA) as standard matrix in order to optimise enzymatic activity and to increase assay linearity, providing a matrix closely resembling human plasma. The assay at hand satisfies common analytical standards of linearity, reproducibility and analyte storage stability. Perspectively, this method is intended to meet the present scientific curiosity around and reveal the role of eNAD^+^ in immunological, oncological and systemic diseases.

## Results

### Evaluation of standard matrix and spectroscopy method

**Figure 1:**
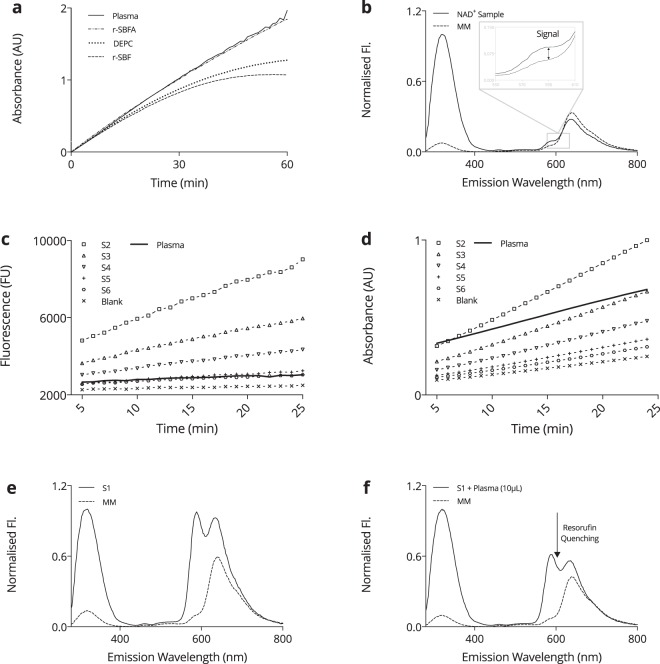
*Overview of the spectroscopic techniques*. (**a**) Comparison of the assay dynamics in different standard matrices utilising the absorbance unit (AU) readings obtained from DEPC water, revised simulated body fluid (r-SBF), revised simulated body fluid adjusted with albumin (r-SBFA) and human plasma, all spiked with 50 μL of 376.8 nM *β*-NAD standard (S2). (**b**) Fluorescence scan of the autofluorescence of the Master Mix (MM) and a NAD^+^ sample as prepared in the actual assay reaction, scanned from *λ*_*ex*1_ = 280 nm − *λ*_*ex*2_ = 850 nm in steps of Δ*λ* = 2 nm. The  resorufin signal is indicated at *λ* = 590 nm in the detail view. (**c**) Enzyme dependent assay kinetics for *β*-NAD standards S1–S6 including a heparinised plasma sample and the blank of a given run using the fluorimetric method. (**d**) Enzyme dependent assay kinetics for *β*-NAD standards S1–S6 including a heparinised plasma sample and the blank of a given run using the colorimetric method. (**e**) Fluorescence scan of the autofluorescence of the Master Mix (MM) and *β*-NAD standard S1 as prepared in the actual assay reaction (S1 + r-SBFA + MM), scanned from *λ*_*ex*1_ = 280 nm − *λ*_*ex*2_ = 850 nm in steps of Δ*λ* = 2 nm. (**f**) Visual representation of the resorufin quenching effect that occurs when a minuscule amount of heparinised plasma sample is added to the *β*-NAD standard S1.

The standard matrix is one of the most crucial components of any assay, as its aim is to match the enzymatic cycling behaviour in plasma to that in the standards. Therefore it is crucial to the validity of the assaying method. In order to emulate the enzyme kinetics of ADH in human heparinised plasma and to gain assay sensitivity, several standard matrices were compared against human plasma, namely DEPC water, r-SBF and r-SBFA, using *β*-NAD Standards. Since a standard matrix might not be completely free of analyte, and, since human plasma endogenously features eNAD^+^, every standard matrix was accompanied by blanks produced from the same matrix. The resulting absorbance unit (AU) readings are presented in Fig. 1a, where all matrices were spiked with 50 μL of *β*-NAD standard S2 (376.8 nM). A qualitative inspection revealed that r-SBFA and heparinised plasma both presented with a highly linear increase, whereas DEPC and r-SBF. The absorbance increase reflects the enzyme velocity of ADH, and was highest in human heparinised plasma, with an increase of approximately 1.95 AU over the course of 60 min and 1.84 AU for the runner up, r-SBFA, concluded by 1.27 AU and 1.07 AU for DEPC and r-SBF, respectively. Analogously, the relative reaction velocity, *v*_*R*_, in plasma and r-SBFA were of remarkable resemblance, namely *v*_*RP*_ = 0.0317 ± 0.0002 and *v*_*RA*_ = 0.0306 ± 0.0002, respectively. All things considered, we found r-SBFA to best emulate *in vivo*-like enzyme kinetics of ADH, therefore adopting it as the standard matrix.

The advantages of fluorimetric over colorimetric detection techniques are well known and include a higher sensitivity and specificity as well as a lower limit of detection. Therefore, we evaluated whether a fluorimetric method was superior to the colorimetric method. For this purpose, MTT was used for absorbance and resazurin for fluorescence, both representing the signal generating molecules. Since the signal of interest arises from the fluorophore ejection from resorufin at a wavelength of 590 nm, an autofluorescence scan of both, the master mix (MM) and a human heparinised plasma sample was performed and a close-up view is given in Fig. 1b. Indeed, the relative autofluorescence spectra of the MM and plasma sample were found to differ only marginally from approximately 0.093 for the eNAD^+^ plasma sample and 0.057 for the MM, yielding a signal to noise ratio (SNR) of 163%. The measured fluorescence unit (FU) and absorbance unit (AU) readings for *β*-NAD standards and human plasma are depicted in Fig. 1c,d. Despite the obvious parallelism of the colorimetric and fluorimetric standard traces of *β*-NAD at different concentrations, the reader is pointed to the apparent nullification of the fluorescence plasma signal. In contrast, the colorimetric detection method exhibited a strong signal for human plasma. In order to elucidate this surprising finding, that is, the apparent resorufin signal quenching in plasma, several autofluorescence scans of the *β*-NAD standards, plasma samples and mastermix (please see methods) were conducted and are given in 1e and 1f. We further explored the fact that a signal was obtainable from the *β*-NAD standards, yet virtually absent from all plasma samples by comparing the autofluorescence spectra of the first *β*-NAD standard S1 (753.6 nM) to S1 spiked with a small (10 μL) quantity of plasma. Strikingly, the relative fluorescence signal at 590 nm was found to be 0.964 for S1 and 0.102 for the MM (945% SNR), which dropped sharply upon the addition of human plasma to 0.614, giving a 602% SNR. Note, the first emission spike around 320 nm from formulations containing either albumin or plasma is in fact due to the autofluorescence of albumin itself, since its tyrosine side chains fluoresces at this specific wavelength^26,27^. Moreover, human blood plasma is known to exhibit substantial autofluorescence around 500–600 nm due to physiologically occurring porphyrins^28–31^.

**Figure 2:**
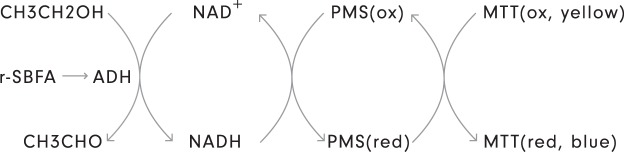
*NAD*^+^ *cycling principle*. Schematic representation of the enzymatic alcohol dehydrogenae (ADH) cycling principle used to measure eNAD^+^, involving phenazine methosulfate (PMS) as the primary and 3-(4,5- dimethylthiazol-2-yl)-2,5-diphenyltetrazolium bromide (MTT) as the secondary redox indicator dye. The method was inspired by the findings of Rhodes *et al*.^20^ and adjusted with insights from the research of Zhu *et al*.^24^ and O’Reilly *et al*.^13^.

In conclusion, we found the colorimetric method, displayed in Fig. 2, superior to the fluorimetric alternative concerning the measurement of eNAD^+^ in human heparinised plasma.

### Reaction time scale and eNAD^+^ storage stability

**Figure 3:**
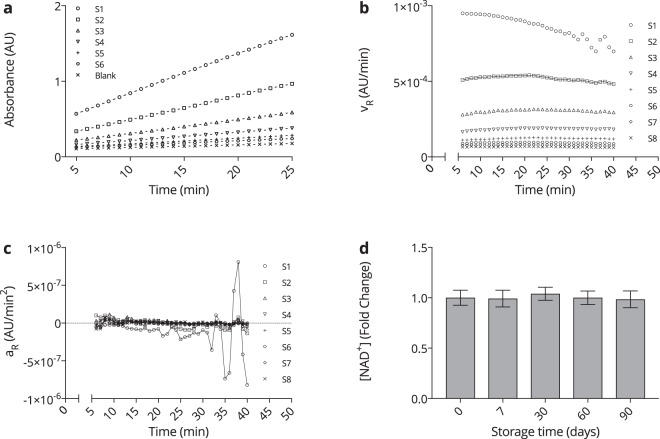
*NAD*^+^ *assay and storage*. (**a**) Enzyme dependent assay kinetics for *β*-NAD standards S1–S6 and the blank of a given run during the assay validation: S1 (753.6 nM), S2 (376.8 nM), S3 (188.4 nM), S4 (94.2 nM), S5 (47.1 nM), S6 (23.5 nM), whilst standards S7 (11.8 nM) and S8 (5.6 nM) were omitted. (**b**) Relative reaction velocities (*v*_*R*_) of standards S1–S8 during min 5–40 of the assay reaction. *n* = 8. (**c**) Relative reaction accelerations (*a*_*R*_) of standards S1-S8 during min 5–40 of the assay reaction. *n* = 8. (**d**) Measured NAD^+^ concentrations in human heparinised plasma stored at −80 °C for a given amount of time. No statistical significance was found when comparing the measured timepoints to the baseline (*d* = 0). *n* = 6. Statistics: Two-tailed, unpaired t-test with the confidence limits of *CL* = 99%, as well as a two-way ANOVA (without repeated measures) adjusted with Tukey’s multiple comparisons test featuring *CL* = 99%. A significance level of *p* < 0.01 was applied to reject the null hypothesis. All error bars are given in terms of ±SD.

To determine the time frame of the assay’s linear operation, the absorbance readings for *β*-NAD standards S1 (753.6 nM) to S6 (23.5 nM) are depicted in Fig. 3a. Clearly, the relative reaction velocity, *v*_*R*_, of S1 decreased, adopting a fluctuating and turbulent behaviour after about min 30, as seen in Fig. 3b,c, with the latter displaying the derivative of *v*_*R*_, *a*_*R*_. From this, we determined the time frame of linear operation between min 5–25 of reaction time.

The stability of eNAD^+^ in human plasma remains an elusive and vague topic of investigation, suffering from a substantial lack of literature. However, eNAD^+^ can be hydrolysed or degraded by multiple enzymes in plasma, such as ADPribosyltransferases (ARTs), as well as NAD^+^ - dependent glycohydrolases (NADases)^32,33^. Likewise, the knowledge of analyte storage stability remains crucial for investigational studies. Therefore, we tested eNAD^+^ storage stability in human heparinised plasma samples at −80 °C over the course of three months, which is displayed in Fig. 3d. Indeed, no statistically significant difference was found between any of the measured time-points, at a significance level of *p* < 0.01, with the measured eNAD^+^ concentration being (225.9 ± 16.7) nM. Hence, eNAD^+^ can be considered stable in frozen human heparinised plasma for at least three months at −80 °C. These results were found to be in accordance with a murine study that demonstrated stability of eNAD^+^ in frozen murine plasma for at least one week and the commercially available *β*-NAD, which was described to be stable for at least six months in aqueous solution^15,34^.

Recapitulating, linear enzymatic behaviour was confirmed for min 5–25 of the assay reaction time and eNAD^+^ was found to remain stable in frozen human heparinised plasma for at least three months.

### Standard linearity and enzyme kinetics

In order to accurately measure eNAD^+^, it is a fundamental requirement that the *v*_*R*_ ratio of any two standards behaves identical to the ratio of their *β*-NAD concentrations. To examine this, the *v*_*R*_ ratio of standard, *n*, and neighbour *n* + 1, was defined to be:

1 $${\gamma }_{n}={v}_{Rn+1}/{v}_{Rn}$$

**Figure 4:**
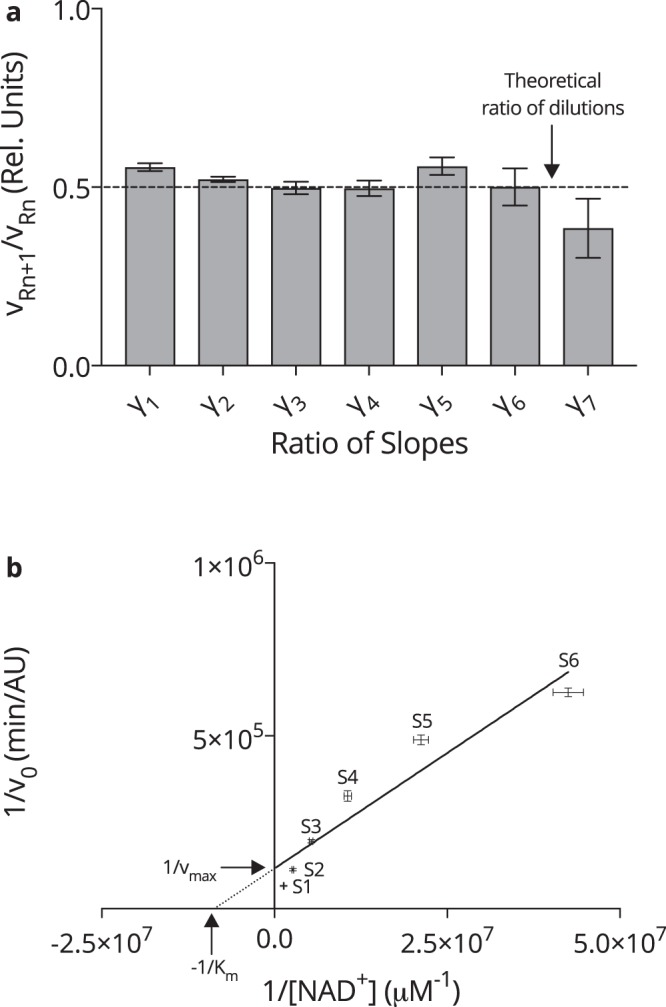
*NAD*^+^ *assay linearity and enzyme kinetics*. (**a**) The ratio of the slope (*v*_*R*_) of two sequential *β*-NAD standards, (*n*) and neighbour (*n* + 1), is given by *γ*_*n*_ between min 5–25 of the assay reaction. *n* = 8. (**b**) The Lineweaver-Burk Plot constructed for averaged, blank corrected, *v*_*R*_ data from eight Runs including S1–S6. *n* = 8. Statistics: All error bars are given in terms of ±SD.

The calculated results are presented in Fig. 4a. One can observe that *γ* remained relatively constant and closely resembled the 50% *β*-NAD standard dilutions for S1 (753.6 nM) to S7 (11.8 nM), with *γ*_1_ = 0.556 ± 0.011 to *γ*_6_ = 0.501 ± 0.052 and corresponding relative errors of ${\delta }_{{\gamma }_{1}}\mathrm{=1.96 \% }$ and ${\delta }_{{\gamma }_{6}}\mathrm{=10.3 \% }$. However, there was an evident drop in the ratio when considering the change from S7 to S8 (5.9 nM), to *γ*_7_ = 0.385 ± 0.083, where the relative error increased to ${\delta }_{{\gamma }_{7}}\mathrm{=21.6 \% }$

Moreover, NAD^+^ was employed as the new substrate in the double reciprocal Lineweaver-Burke plot^35^. The data displayed in Fig. 4b was constructed from eight independent assay reactions, where the Michaelis-Menten constant was found to be *K*_*m*_ = (115 ± 45.6) nM in addition to the maximum reaction velocity being ${v}_{{\max }}=(8.58\pm 3.13)\,{\rm{\mu }}\mathrm{mol}\,{{\rm{\min }}}^{-1}$.

In summary, the linear relationship between the standard slopes (*γ*) was confirmed for S1 (753.6 nM) down to S7 (11.8 nM), covering sufficiently the range of anticipated physiological concentrations of eNAD^+^^13^.

### Reliability of NAD^+^ detection in relation to albumin

Physiologically, serum albumin is the most abundant protein of human plasma. Patients with liver diseases can suffer from hypoalbuminaemia, presenting with levels below 35 g/L and drastic inter- as well as intrapatient variability, while healthy subjects typically feature levels in the range of 35–50 g/L. Hence, we studied the effect of varying albumin concentrations on the assay’s predictive capability. As illustrated shortly, an ordinary regression line that runs not through the origin (nTTO, *v*_*R*_ = *mx* + *v*_*b*_), as opposed to a regression line through the origin (TTO, ${v}_{R}^{^{\prime} }$ = *mx* + 0), is subject to an inversely proportional relationship between the albumin concentration of the matrix and the calculated eNAD^+^ value, rendering it unworkable for analysis.

**Figure 5:**
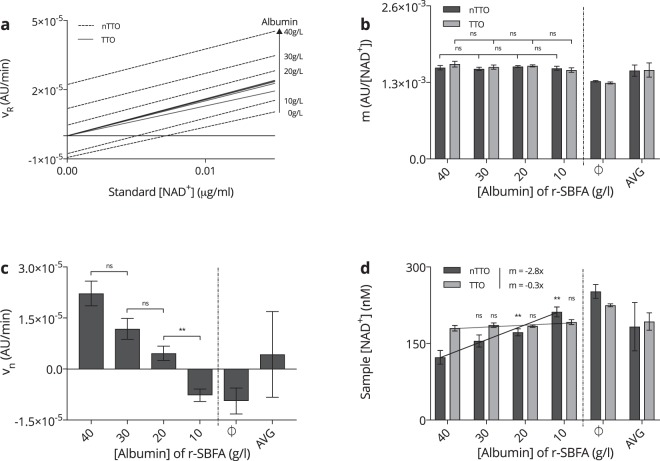
*Albumin dependency of the assay*. (**a**) Comparison of the calibration working curves not through the origin (nTTO, dotted) and to through the origin (TTO, solid) with respect to a varying albumin concentration in the *β*-NAD standards S1 (753.6 nM) through S6 (23.5 nM) of 0 g/L for the lowermost trace, 10 g/L, 20 g/L, 30 g/L and 40 g/L for the uppermost trace. *n* = 3. (**b**) Comparison of the slopes, *m*, of the calibration working curves obtained by linear regression analyses nTTO (dark) and to TTO (light) for different albumin concentrations. *n* = 3. (**c**) Variance of the y-axis intercept, *v*_*n*_, of the regression lines nTTO with respect to varying albumin concentrations. *n* = 3. (**d**) Predicted pooled human heparinised sample eNAD^+^ concentrations using calibration working curves constructed nTTO (dark) and to TTO (light) with respect to varying albumin concentrations. A regression fit over the physiological range of albumin concentrations yielded *y* = −2.84 *x*nML/g + 236.5 nM (*R*^2^ = 0.9785) for the nTTO and *y*′ = −0.34 *x*nML/g + 194 nM (*R*^2^ = 0.7706) for the TTO approach. *n* = 3. Statistics: Two-tailed, unpaired t-test with the confidence limits of *CL* = 99%, where a significance level of *p* < 0.01 was applied to reject the null hypothesis. **p < 0.01. All error bars are given in terms of ±SD. The dotted line represents the physiological range of albumin concentrations.

In order to demonstrate this, we evaluated various r-SBFA matrices featuring 0 g/L (r-SBF), 10 g/L, 20 g/L, 30 g/L and 40 g/L of albumin. In fact, one can observe from Fig. 5a, where nTTO and TTO calibration curves are contrasted, that their slopes remained relatively constant with varying concentrations of albumin for $m=(1.50\pm 0.10)\,{{\rm{ngAUmL}}}^{-1}\,{{\rm{\min }}}^{-1}$ and $m^{\prime} =(1.51\pm 0.12)\,{{\rm{ngAUmL}}}^{-1}\,{{\rm{\min }}}^{-1}$, respectively. The individual values for the slopes of the nTTO and TTO approach are displayed more precisely in Fig. 5b. Here, no statistically significant differences between the neighbouring slopes of the physiological range of albumin concentrations (10–40 g/L) was found. However, Fig. 5a clearly demonstrates that an increase in the albumin concentration in the standard matrix caused an upward shift of the y-axis intercept, *v*_*b*_, for lines constructed with a nTTO approach and is numerically presented in Fig. 5c, where the baseline can be seen to vary from ${v}_{b0}=(\,-\,9.40\pm 3.81)\,{{\rm{pgAUmL}}}^{-1}\,{{\rm{\min }}}^{-1}$ for no albumin (0 g/L) up to ${v}_{b40}=\mathrm{(4.31}\pm \mathrm{12.6)}\,{{\rm{ngAUmL}}}^{-1}\,{{\rm{\min }}}^{-1}$ for 40 g/L of albumin, with an average value of ${v}_{b}=(4.31\pm 12.6)\,{{\rm{ngAUmL}}}^{-1}\,{{\rm{\min }}}^{-1}$, indicating a relative error of ${\delta }_{{v}_{b}}=293 \%$. No significant difference was found between *v*_*b*40_ and *v*_*b*30_ (*p* = 0.0193) as well as *v*_*b*30_ and *v*_*b*20_ (*p* = 0.0289), while *v*_*b*20_ and *v*_*b*10_ were, in fact, significantly different (*p* = 0.0016). Figure 5d reveals that the TTO method resulted in an average sample NAD^+^ concentration of *x*′ = 193 ± 17.0 nM, indicating a relative error of *δ*_*x*′_ = 8.8% in contrast to *x* = 183 ± 47.4 nM for the nTTO approach, presenting with an escalated relative error of *δ*_*x*_ = 25.9%. Moreover, the TTO approach was fitted with an ordinary least squares regression, with CL = 99%, to produce *y*′ = −*x*0.34 nML/g + 194 nM (*R*^2^ = 0.7706), whilst the nTTO method featured *y* = −*x*2.84 nML/g + 237 nM (*R*^2^ = 0.9785), representing an approximately 8 times higher dependence of estimated eNAD^+^ on albumin. When measuring the estimated eNAD^+^ amounts, the TTO method far outperformed the nTTO method. Merely a concentration of 30 g/L of albumin let to an insignificant increase of the estimated eNAD^+^ (*p* = 0.0361), whilst 20 g/L (*p* = 0.0049) and 10 g/L (*p* = 0.0007) of albumin caused significantly different predicted eNAD^+^ results. On the other hand, this effect was dampened when the TTO method was used as no significantly different estimated eNAD^+^ concentrations were quantified at any of the given albumin concentrations, since *p* < 0.01.

Taken together, differing serum albumin levels cause inaccurate estimations of sample eNAD^+^ concentrations when the nTTO method is used. Strikingly, this effect was virtually eliminated, when calibration was constructed TTO, which lead to a substantially lower relative error as well as albumin related estimation bias.

### Reproducibility, Sensitivity and Calibration

**Figure 6:**
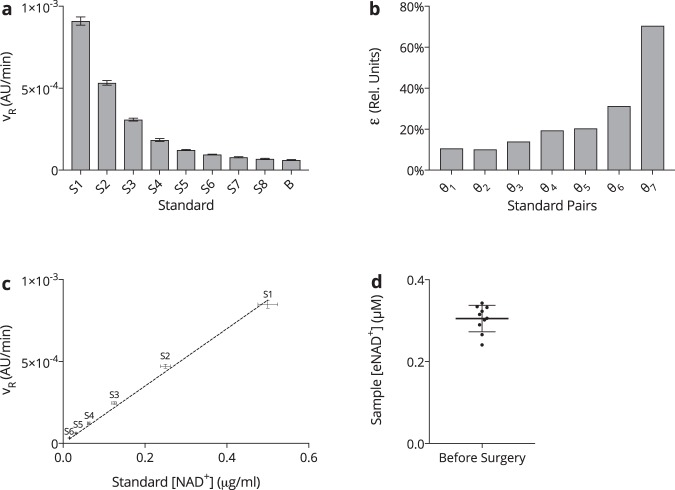
*NAD*^+^ *assay reproducibility*. (**a**) Mean relative reaction velocities (*v*_*R*_) for *β*-NAD standards S1 (753.6 nM) through S8 (5.6 nM) and the blank measured between min 5–25 of the assay reaction. *n* = 8. (**b**) The ratio (*ε*_*n*_) of standard deviations (*σ*_*n*_), to the difference in relative reaction velocity (*v*_*R*_) of two sequential *β*-NAD standards, (*n*) and neighbour (*n* + 1), represented by *ψ*_*n*_. *n* = 8. (**c**) Calibration working curve constructed from the average slopes (*v*_*R*_) of *β*-NAD standards S1 (753.6 nM) through S6 (23.5 nM), obtained from the eight-fold assay repetition. *n* = 8. (**d**) Blood samples were taken from patients scheduled to undergo hernioplasty and analysed for their eNAD^+^ concentration. Statistics: *n* = 10. Among the analysed, there were 8 male and 2 female patients, where the mean age averaged (52.1 ± 15.8) years, ranging from 29 to 75 years. The underlying diseases were inguinal hernia (*n* = 7), epigastric hernia (*n* = 1), hiatal hernia (*n* = 1) and umbilical hernia (*n* = 1). The overall average is displayed. Statistics: Two-way ANOVA (without repeated measures) adjusted with Tukey’s multiple comparisons test with CL = 99%. A significance level of *p* < 0.01 was applied to reject the null hypothesis. ****p < 0.0001. All error bars are given in terms of ±SD.

For the sake of evaluating the repeatability and robustness of the assay, the complete method was independently carried out eight times, with the respective slopes of the standard curves calculated within the interval of min 5–25 of the reaction time. The corresponding results are given in terms of the relative reaction velocity for each *β*-NAD standard *n*, *v*_*Rn*_, and are displayed in Fig. 6a. In descending order the relative reaction velocities of standards S1 (753.6 nM) to S8 (5.9 nM) as well as the blanks were determined to be *v*_*R*1_ = (9.10 ± 0.28) × 10^−4^ AU/min for S1 down to *v*_*RS*8_ = (6.84 ± 0.42) × 10^−5^ AU/min for S8 and *v*_*RB*_ = (6.18 ± 0.33) × 10^−5^ AU for the blank. The use of a two-way ANOVA (without repeated measures), adjusted with Tukey’s multiple comparisons test, revealed that there was no significant difference observable between S6 (23.5 nM) and S7 (11.8 nM, *p* = 0.012), S7 and S8 (5.9 nM, *p* = 0.373), S7 and the blank (*p* = 0.013), or S8 and the blank (*p* = 0.876). In order to examine the potential implications for the precision of the assay when an additional *β*-NAD standard, *n*−1, was used for calibration, the ratio of the standard deviation, *σ*, to the difference in *v*_*R*_, defines the introduced resolution error, *ε*:

2 $${\varepsilon }_{n}=\frac{{\sigma }_{n}+{\sigma }_{n+1}}{{v}_{Rn}-{v}_{Rn+1}}$$

The respective values for *ε* are depicted in Fig. 6b, where the resolution error introduced by the consideration of S1 through S6 was found to be below *ε*_1–5_ < 20%, whereas the inclusion of S7 (11.8 nM) and S8 (5.9 nM) lead to an escalated resolution error of *ε*_6_ = 30% and *ε*_7_ = 70%, respectively. Having defined a threshold value of *ε* = 20%, S7 and S8 were discarded from the assay for their lack of a statistically relevant *v*_*R*_ and inflation of *ε*.

Following this discovery, an ordinary least squares linear regression fit of the blank adjusted standard calibration curve was constructed from S1–S6 to run through the origin (TTO), shown in Fig. 6c, where *ρ* = 0.9984 and *R*^2^ = 0.9969, with the corresponding slope $m=(1.75\pm 0.04)\,{{\rm{ngAUmL}}}^{-1}\,{{\rm{\min }}}^{-1}$.

In order to present clinical findings of eNAD^+^ concentrations in blood plasma, we obtained heparinised blood samples from 10 fasting patients, who were scheduled to receive hernioplasty. Subsequent to obtaining the plasma samples, we determined the eNAD^+^ value to have a mean concentration of (305.2 ± 32.2) nM (range: 240.9–342.7 nM). The graphical representation of this data is given in Fig. 6d. What is more, the analysis of one samples that was carried along all of the eight runs, resulted in a relative error of *δ* = 8% in the measured eNAD^+^ concentration.

When combining these results, the method was able to sense eNAD^+^ in human heparinised plasma and in standards featuring concentrations of 753.6 nM (S1) down to 23.5 nM (S6) maintaining a striking calibration precision yet exhibiting a minor relative error.

## Discussion

In this study, we established a reliable and robust colorimetric two-step enzymatic cycling assay to quantify eNAD^+^ in human heparinised plasma, which can be used for high throughput screening of eNAD^+^ levels in clinical discovery studies. The assay quantifies eNAD^+^ by means of a two-step enzymatic cycling reaction, based on ADH (Fig. 2). An albumin modified r-SBF was used to warrant physiological enzymatic activity. In addition, we evaluated assay linearity, reproducibility and confirmed long-term storage stability of eNAD^+^ in frozen human heparinised plasma.

However, the correct blood collection method is of the essence. We employed lithium heparin tubes during the acquisition of blood samples as the common chelating agent K3EDTA is in fact a potent inhibitor of ADH, and would therefore eradicate linear enzyme behaviour^36^. In order to secure optimal enzyme activity, the concentrations of ADH as well as its substrate, ethanol, were chosen to be well beyond their point of saturation, so that any effect of even an ever so slight variation in their respective amounts would be essentially nullified^24^.

In order to validly calculate eNAD^+^ levels in human plasma, it was imperative to employ an appropriate and *in vivo*-like standard matrix. Physiologically speaking, albumin is the most abundant plasma protein, with a concentration in the range of 35–50 g/L, whilst being the main carrier of zinc in blood, as approximately 80% of all plasma zinc is bound to albumin^37^. Thus it serves as a major zinc donor in blood, comprising part of the so called exchangeable pool, which, given zinc’s role as a blood trace element, is crucial to the proper functioning of ADH. In fact, a catalytic zinc ion binds to ADH’s Cys-43, Cys-153 and His-66 amino acid residues, which was the primary rationalisation for our addition of albumin into r-SBF, since zinc is neither present in r-SBF, nor in DEPC^38^. This could very well explain the evident deviation from linearity after about 30 min in all zinc-free matrices, indicating a decreased ADH reaction speed and successive plateauing substrate conversion rate. However this was alleviated through the addition of 40 g/L of albumin to r-SBF, as demonstrated in Fig. 1. Indeed, we hypothesise that, under the assumption that albumin’s affinity for zinc is not infinitely large, some of these zinc ions will be competed for, and eventually be seized by ADH. In addition, the extreme pH milieus, combined with the heat incubation upon pyridine extraction, will cause a release of zinc from albumin, facilitated by its denaturation. Results obtained from the comparison of different standard matrices, presented in Fig. 1a, seem to corroborate this hypothesis, for r-SBFA replicated the assay kinetics observed in human heparin plasma almost indiscriminately.

Moving on to the analyte isolation, a heat based dichotomous pH extraction procedure was utilised to extract and amass purified versions of either NAD^+^ at pH 1.5 or NADH at pH 12.5, as can be seen in Table 5, in consideration of Lowry *et al*. and Lilius *et al*.^18,25^. An illustration of this process is given in Supplementary Fig. S.2. As the assaying method was intended to quantify eNAD^+^ plasma levels in exploratory studies, it was necessary to test storage stability of eNAD^+^ in human heparinised plasma at −80 °C. This was done to evaluate the possibility, that eNAD^+^ would be hydrolysed by ectoenzymes from the family of NADases such as CD38 or ADPribosyltransferases like ART2^5,39^. Subsequently, we confirmed the stability of eNAD^+^ in frozen human plasma for at least three months, which was in accordance with a murine study that demonstrated its stability for at least one week, as well as the known stability of *β*-NAD in aqueous solution for at least six months^15,34^.

With respect to the fluorimetric evaluation as an alternative quantification method, the reader is pointed to some potentially detrimental properties of human blood plasma that hinder the measurement of eNAD^+^ using resazurin. First and foremost, naturally occurring porphyrins cause the autofluorescence of human blood plasma to exhibit two prominent spikes at 630 nm and 590 nm, of which the latter one happens to be at the exact frequency resorufin fluoresces at^28^. It is further known that these autofluorescence spectra can vary substantially over the range of 500–600 nm, due to immense variations in plasma porphyrin levels, which are common in patients with cancer^29–31^. What is more, another biological molecule with a broad autofluorescence band around 500–600 nm is bilirubin^40^. The vast fluctuations in bilirubin levels, especially in patients suffering from liver disease can therefore cause significant baseline shifts that are very difficult to correct for. Having used resazurin instead of MTT, we immediately realised the almost complete eradication of the signal in plasma, whilst the standards remained readily quantifiable. Moreover, we tested the impact of adding a small amount of a plasma sample to the first *β*-NAD standard, S1, upon which its obtained signal experienced a sharp drop, clearly demonstrated in Fig. 1e,f. Besides the aforementioned reasons, a few possible explanations are herein presented that could be responsible for this apparent quenching of the resorufin signal at the 590 nm emission band. In fact, Montejano *et al*.^41^ described that resorufin is quenched by quinones at this very frequency band^41^. Given the endogenous nature of these molecules, the sharp drop in resorufin’s fluorescence upon the addition of a small plasma quantity (which even contained physiological eNAD^+^ concentration) supports this hypothesis and explains why eNAD^+^ is not measurable in a full plasma sample via fluorescence spectroscopy.

In comparison to other measuring procedures of NAD^+^, it has to be stated that eNAD^+^ has been studied in a far lesser extent than iNAD^+^, most likely for its drastically reduced concentration, from 10–40 M intracellularly to 240–290 nM extracellularly, which presents nontrivial analytical challenges^11–13^. Alternative to the quantification of chromogenic or fluorescent signals, the concentration of iNAD^+^ has been measured using a multitude of sophisticated high-performance liquid chromatography (HPLC) methods such as HPLC-UV^42,43^, as well as HPCL-NMR^44,45^, whilst, in a more specialised scenario, HPLC-UV^11,46^ and HPLC-MS^47,48^, have been employed to evaluate murine erythrocyte intracellular NAD^+^ (iNAD^+^) levels. These methods have been used predominantly to measure iNAD^+^, while only very few could be used to study eNAD^+^ directly without any preceding concentrating steps, for their relatively high limit of quantification. However, Liang *et al*.^15^ used a modified version of the HPLC-MS, namely the electrospray ionisation HPLC-ESI-MS to successfully measure eNAD^+^ in murine blood^15^. Nonetheless, for all analytical methods involving calibration methods, the standard matrix has to exhibit comparable traits to the authentic biological sample matrix and should either be a laboratory prepared or analyte-stripped version of the matrix of interest. In fact, Liang *et al*.^15^ used HPLC grade water in their HPLC-ESI-MS analysis, which seemed to be common practice amongst other HPLC based analyses^15^. This opens up the analysis of NAD^+^ to be subject to significant signal distortions through imperfect enzyme kinetics, signal masking by other analytes or by means of ionisation suppression^14^. In addition, the apparatus required for HPLC analyses tends to be rather expensive, therefore limiting their availability to the general scientific community, whereas our enzymatic cycling method merely required an ordinary microplate reader, present in virtually all laboratories by default, in addition to any kind of transparent 96 well plate.

When it comes to plasma compounds that could affect the assays performance, we postulate that ADH is the main source of measurement variation, as it represents the main rate limiting factor in the redox dye conversion reaction. For instance, human isoforms of ADH are generally inhibited by common drugs such as aspirin or H2 receptor blockers in the form of cimetidine or ranitidine^49,50^. These drugs might be present in the plasma of individual patients at distinct concentrations. What is more, these drugs might very well resist, in part, the extraction, neutralisation and deproteinisation steps, thereby modulating the activity of ADH in the assay reaction. This could cause eNAD^+^ measurement variations at an inter- as well as intrapatient levels. ADH is characterised further through a modulatory effect by different hormones, such as growth hormones, epinephrine or estrogens which act in a stimulatory manner, while thyroid hormones^51^ and androgens can inhibit ADH’s activity^52,53^. Therefore, varying baseline levels of these hormones upon blood collection could further impact the behaviour of ADH in the plasma sample. However, this assumption seems to be more theoretical as the extraction step (60 heat incubation for 10 min at pH 1.5) is expected to denature virtually all proteins and hormones. It is well known that ADH features broad specificity to aliphatic alcohols other than ethanol. For instance, it oxidises methanol to produce formaldehyde. In fact, methanol is of varying concentration in human blood, partly due to dietary preferences for artificial sweeteners such as aspartame^54^. Fasting blood levels of methanol were found to be on average 168 μmol/L, which converts to 5.39 mg/dL^55^. However, the assay procedure will cause a 6-fold dilution of methanol levels, yielding an approximate concentration 1 mg/dL, which is less than 1% of the ethanol concentration in the MM (being 125 mg/dL). Furthermore, ADH can also metabolise retinol (Vitamin A), which is present in blood plasma at levels around 60 μg/dL^56^. It is due to the fact that ethanol’s concentration, as key substrate of ADH in the MM, was chosen to lie well beyond the point of saturation, that neither methanol nor retinol are expected to have a prominent effect on the measured eNAD^+^, for their minuscule concentrations. In summary, we hypothethise that hormones, proteins and vitamins endogenous to human blood, will produce merely negligible effects on the assay, thanks to the selection of our extraction and neutralisation procedure, which provides an immensely harsh environment that most of these compounds cannot withstand.

The presented method was exhaustively tested in human heparinised plasma and the analysis as well as the experimental protocol, attached to this article as Supplementary Method S.1, account for immense variations in the standard and sample matrices, due to albumin fluctuations. In respect of the findings displayed in Fig. 5, it was evident that a regression line TTO would be used for all measurements, as it first and foremost increased the robustness of the method and secondly it was simply not feasible to produce a tailored albumin blank for every human heparinised plasma sample due to practical reasons. Moreover, albumin concentrations are rarely known, as they are not covered by standard blood chemistry tests. This, is as the evidence gathered and presented in Fig. 5a,c, illustrates the high dependency of the y-axis intercept, *v*_*b*_, of the assay’s nTTO calibration curve on the albumin concentration of the standard matrix. Hence, when utilising those calibration curves to estimate the eNAD^+^ of an actual plasma sample, one will obtain very uncertain and unreliable results, as *v*_*b*_ in the equation of the regression line, when extrapolating for an unknown amount, *x*, takes the role as a subtractor of *v*_*R*_, and, given that we are dealing with relatively small values of *m* in the denominator, causes escalated fluctuations of the resulting value. On a critical note, an estimation of the random error that arise when preparing the standards and the total random error, including the final addition of the MM to the standard, are given below. For this purpose, the common propagation of uncertainty analysis was conducted, where pipetting as well as applicable preparation errors, such as weighing scale or glassware uncertainties, were considered. For any given measurement of the experimental factors *a*, *b* and *c* resulting variable, *Q*, that is the combination of sums and differences, *a* + *b*, *Q* = *a* + *b* − *c*, the uncertainties, Δ, add in quadrature, that is;

3 $${\rm{\Delta }}Q=\sqrt{{({\rm{\Delta }}a)}^{2}+{({\rm{\Delta }}b)}^{2}+{({\rm{\Delta }}c)}^{2}}$$

On the other hand, if *R*, is the resulting variable of a multiplication or division, $R=\frac{a+b}{c}$, then the fractional uncertainties add in quadrature;

4 $$\frac{{\rm{\Delta }}Q}{Q}=\sqrt{{(\frac{{\rm{\Delta }}a}{a})}^{2}+{(\frac{{\rm{\Delta }}b}{b})}^{2}+{(\frac{{\rm{\Delta }}c}{c})}^{2}}$$

In fact, this resulted in the estimation of the random relative error in preparing S1 of *δ*_*S*1_ = 4.84%, increasing to *δ*_*S*6_ = 5.20% for S6, while the total relative error after the addition of the MM for S1 was determined to be *δ*_*T*1_ = 8.29%, increasing to *δ*_*T*6_ = 8.50% for S6. Obviously, this error can be minimised by preparing reagents in aliquots, where possible.

On a practical note, experimenter is able to perform the whole method given in Supplementary Method S.1, in approximately 90 min. When employing a commonly used 96 well plate this would imply a total experiment time of roughly 1 min per sample, representative of a substantial increase in time efficiency when compared to the commonly employed HPLC quantification methods, that usually require 10–60 min of reaction time per sample analysed.

At a molecular level, eNAD^+^, has been identified to be of intricate involvement in a substantial number of regulatory pathways as a coenzyme for ADPribosyltransferases (ARTs), NAD^+^-dependent protein deacetylases of the Sir2 family (SIRTs) as well as NAD^+^-dependent glycohydrolases (NADases), in addition to serving as a precursor of the calcium mobilising molecule cADPR (cyclic ADP-ribose)^32,33,57^. The highly modulating properties of eNAD^+^ are, on this note, underscored by its substantial involvement in highly specific and selective ectoenzymes, namely NADases such as CD38, ADPribosyltransferases like ART2 or its key role in purinergic signalling through P2X7 or P2Y^5,39,58^. Leading on to the systemic involvement of eNAD^+^ in immunological processes and its relevance to human physiology, the plasma membrane of eukaryotic cells was long believed to be impermeable to NAD^+^. Yet, after some initial findings of passive pyridine nucleotide transmembrane-transport, Bruzzone *et al*.^59^, successfully identified this elusive NAD^+^ transporter as the hexametric hemichannel Cx43^59^. Cx43 is a ubiquitous transmembrane protein, that, when juxtaposed on adjacent cells, forms local high density areas at the gap junctions between these cells^60^.

In conclusion, we herein present a robust method for the measurement of eNAD^+^ in human heparinised plasma standardised, validated and optimised for low-cost high throughput screening applications using off-the-shelf materials, including standard laboratory equipment such as microplate readers. This method was established to help validate the future resumption of our working hypothesis being that eNAD^+^ is involved in extracellular paracrine signalling with implications for various systemic diseases. Moreover, our method facilitates the translational research of eNAD^+^ and its role in aging as well as the pathogenesis of oncologic or other systemic diseases by lowering the economic barriers of expensive experimental devices.

## Methods

### Establishment of the cycling method

The protein alcohol dehydrogenase (ADH, EC1.1.1.1) from Saccharomyces cerevisiae was employed to measure the pyridine nucleotides NAD^+^ and NADH, due to its specificity for NAD^+^ as a coenzyme, which, given the cycling nature of the application promotes NADH to the status of being a coenzyme as well^61^. Illustratively, a schematic of the principle behind this cycling assay is provided in Fig. 2, where the reader can follow the path of the electrons which are transferred during one cycle of the assay. Initially, the NAD^+^ dependent dehydrogenase ADH catalyses the conversion of ethanol into acetaldehyde (ethanal)^20^, reducing its coenzyme NAD^+^ to NADH in the process. In turn, this reduced pyridine nucleotide donates an electron to the secondary redox indicator dye, 3-(4,5-dimethylthiazol-2-yl)-2,5-diphenyltetrazolium bromide (MTT), via a preceding coupled reaction with the primary indicator dye, phenazine methosulfate (PMS). The reduction of MTT, and subsequent formation of formazan, can then be assayed colorimetrically and linked to the concentration of NAD^+^ (and NADH).

In order to distinguish NAD^+^ from NADH, the impeccable works of Lowry *et al*.^62^ on ‘The Stability of Pyridine Nucleotides’, provided us with a method of extracting the respective nucleotides by means of heat-incubating them in different pH milieus. Thereby, NAD^+^ could be extracted through the addition of a strong acid, destroying all NADH molecules, whereas NADH is extracted with a strong base, destroying all NAD^+^ molecules. The extraction steps were chosen to feature pH 1.5 for the NAD^+^ extraction and pH 12.5 for the NADH extraction. In order to enzymatically measure the respective pyridine nucleotides, the samples were neutralised at pH 7.4 in order to shift the pH into the physiological working range of ADH. The role of r-SBFA in this method was to emulate heparinised blood plasma as the standard matrix.

### Preparation of chemicals

The assay reaction was initialised, and thus the signal was induced through a Master Mix (MM), which, apart from NAD^+^, contained all the molecules required for the cycling reaction which consisted of the following reagents: Alcohol Dehydrogenase from yeast in suspension, EC1.1.1.1 (ADH, SIGMA, USA, Catalogue No: 10127558001), thiazolyl blue tetrazolium bromide (MTT, Sigma, USA, Catalogue No: M2128), phenazine methosulfate (PMS, Sigma, USA, Catalogue No: P9625), ethanol (100%), Triethanolamine (TEA, Sigma, USA, Catalogue No: 90279), and diethyl dicarbonate (DEPC, Sigma, USA, Catalogue No: D5758) water. As employed in the MM, the TEA Buffer, as well as the ADH solution were prepared in a ten-fold dilution with DEPC water, whilst PMS was prepared as a 10 mg/mL solution and MTT as a 1 mg/mL solution in DEPC water. In Table 1, a list of materials is given, featuring the molar make-up of the MM and the respective quantities of the molecules employed for a single well (150 μl of MM) and extended to an entire plate (extended to 105 wells, to facilitate the use of a pipetting basin). Upon the evaluation of a fluorimetric method, MTT was substituted with resazurin (Sigma, USA, Catalogue No: R7017).

**Table 1:** List of materials used for the preparation of 150 μL master mix per well, scaled to fit a standard 96 well plate (where 105 wells are considered necessary for the use of a pipetting basin). Note that the reagents employed are the products of this protocol so far, and have thus been preciously diluted and weighed into DEPC water.

Reagent	Q (μmol)	Well (μL)	Plate (μL)
TEA	25.72	33.6	3528
EtOH	129	7.5	788
PMS	0.4	12.3	1292
MTT	0.1	41.4	4347
DEPC	—	41.1	4316
ADH	125 U	14.4	1512

The NAD^+^ and NADH extraction and neutralisation buffers were then prepared in DEPC water, where the NAD^+^ extraction buffer consisted of a 0.3 N HCl solution and the neutralisation buffer comprised equal parts of 0.36*N* TEA-HCl (ACROS, USA, Catalogue No: 170051000) and 0.6*N* KOH. Analogously, 0.3N KOH was used for the NADH extraction buffer, whilst the neutralisation buffer was composed of 23% 0.36N TEA-HCl, 23% 0.6N HCl and 54% DEPC water. PMS and MTT stock solutions were stored in aliquots at −20 °C until use. The MM was prepared freshly just prior to measurement to prevent auto-oxidation of PMS and MTT as well as a denaturation of ADH.

### Preparation of stock solutions

The linearity, sensitivity, and specificity of the assay was determined with *β*-NAD (Sigma, USA, Catalogue No: N6522) as a reaction standard used for the construction of calibration curves. Firstly, a 1 mg/L stock solution of *β*-NAD in DEPC water was prepared, followed by a thousand-fold dilution in DEPC water. Successively the first calibration standard (S1), was obtained to entail a concentration of 0.5 μg/mL (753.6 nM) by a further one to one dilution of 500 μL in 500 μL DEPC. Afterwards, 5 additional serial dilutions constituting 500 μL of previous Standard in 500 μL DEPC water were performed to obtain S2 (376.8 nM) through S6 (23.5 nM), respectively, forming a total of six calibration standards, which are summarised in Table 2. Initially, two further dilutions were prepared as S7 with a concentration of 11.8nM and S8 with a concentration of 5.9 nM, however these were excluded from the assay upon evaluation of their suitability as calibration standards. Until use, prepared *β*-NAD aliquots were stored at −80 °C.

**Table 2:** List of *β*-NAD standards prepared in DEPC water with respective concentrations.

Standard	*β*-NAD (n*M*)	*β*-NAD (ng/mL)
S1	753.6	50.00
S2	376.8	25.00
S3	188.4	12.50
S4	94.2	6.25
5	47.1	3.13
6	23.5	1.56

### Standard matrix preparation

When operating enzymatic assays, and subsequent sample concentration extrapolation using calibration curves, it is essential to carry the standards (S1–S6) in a matrix that adequately reflects and resembles the physiological properties of human blood plasma, the *in vivo* matrix of eNAD^+^. Note that, deriving a plasma based standard matrix completely free of NAD^+^ is not feasible because of the endogenous nature of this analyte. For this reason, several commonly used simulated body fluids (SBF) were theoretically compared to blood plasma based on their ionic composition and buffering capabilities, and are displayed in Table 4^63^. Due to its remarkable ionic resemblance to blood plasma, we employed a revised simulated body fluid (r-SBF) to serve as the standard matrix. In addition to that, r-SBF contained HEPES buffer, which features an acid dissociation constant of *p*_*ka*_ = 7.5, thus more closely resembling the buffering nature of plasma than the otherwise commonly used Tris buffer, exhibiting *p*_*ka*_ = 8.07. Although the process for finding this standard matrix is presented and discussed in the following sections, it was found that the addition of albumin has proven crucial in emulating the assay’s enzyme kinetics in human plasma. Finally, a list of materials used to prepare 1000 ml of a revised simulated body fluid adjusted with albumin (r-SBFA) in DEPC water is given in Table 3, below. In fact, the albumin concentrations of the r-SBFA were evaluated with respect to their impact upon the assay performance at 0 g/L (r-SBF), 10 g/L, 20 g/L, 30 g/L and 40 g/L. The prepared r-SBFA was stored in aliquots at −20 °C, until use.

**Table 3:** List of materials used for the preparation of 1000 mL of a revised simulated body fluid adjusted with albumin (r-SBFA) where the pH was adjusted to be 7.5, using approximately 0.8 mL 1.0 N NaOH. The composition of the r-SBF without albumin was adopted from Oyane *et al*.^63^.

Reagent	Supplier	Amount (g)
NaCl	SIGMA, USA, Catalog No: DE71376	5.403
NaHCO_3_	SIGMA, USA, Catalog No: DES5761	0.740
Na_2_CO_3_	SIGMA, USA, Catalog No: DES7795	2.046
KCl	ROTH, GER, Catalog No: 6781.1	0.225
KH_2_PO_4_	ROTH, GER, Catalog No: 3904.1	0.138
MgCl_2_⋅6H_2_O	ROTH, GER, Catalog No: HN03.1	0.311
HEPES	ROTH, GER, Catalog No: 9105.4	11.928
CaCl_2_⋅2H_2_O	ROTH, GER, Catalog No: HN04.1	0.388
Na_2_SO_4_	SIGMA, USA, Catalog No: S6547	0.072
BSA	SERVA, GER, Catalog No: 47330.03	40

**Table 4:** Common simulated body fluids compared to human blood plasma, where ionic concentrations are given in mM. The table was extracted from the findings of Oyane *et al*.^63^.

Formulation	Na^+^	K^+^	Mg^2+^	Ca^+^	Cl^−^	HCO_3_^−^	HPO_4_^−^	SO_4_^−^	Buffer
Blood Plasma	142.0	5.0	1.5	2.5	103.0	27.0	1.0	0.5	—
Original SBF	142.0	5.0	1.5	2.5	148.8	4.2	1.0	0.0	Tris
Corrected (c-SBF)	142.0	5.0	1.5	2.5	147.8	4.2	1.0	0.5	Tris
Revised (r-SBF)	142.0	5.0	1.5	2.5	103.0	27.0	1.0	0.5	HEPES
Modified (m-SBF)	142.0	5.0	1.5	2.5	103.0	10.0	1.0	0.5	HEPES

### Blood sample collection

Human peripheral venous blood from surgical patients, approved by the Charité ethics committee (Ethikkommission der Charité Universitätsmedizin Berlin, EA1/291/17 and EA1/018/17), was collected - after having obtained informed consent - into lithium heparin, complying with local regulatory guidelines and the Declaration of Helsinki. Samples were promptly centrifuged at 2500 g for 15 min at 4 °C in order to separate the plasma from all corpuscular parts of the blood, which was successively collected and snap frozen in liquid nitrogen. The resulting plasma aliquots were then transferred to a −80 °C freezer, where they were stored until being assayed for eNAD^+^ concentrations. In addition to our own storage time evaluation, literature provided sufficient evidence that eNAD^+^ in frozen plasma, as well as the commercially available *β*-NAD, used in this method, were stable under these conditions^15,34^.

### NAD extraction protocol

As previously described, we based the extraction procedure of NAD^+^ and NADH on the findings of Lowry *et al*.^18^, adjusted with insights from Lilius *et al*.^25^ and O’Reilly *et al*.^13^. Heparin plasma samples were treated with 0.3N HCl to extract NAD^+^ and 0.3N KOH to extract NADH, following their collection from the −80 °C freezer and after subsequent thawing at room temperature. This is since NAD^+^ is known to be stable for at least half an hour at room temperature^15^. Initially, 300 μL of sample were transferred into a new tube for NAD extraction and 30 μL sample + 270 μL r-SBFA for NADH extraction. Upon addition of the acid and base solutions for pyridine nucleotide extraction, the samples were vortexed and then incubated at 60 °C for 10 min. Following this, the samples were promptly equilibrated on ice for 10 min. Subsequently, the samples were neutralised so as they would exhibit ADH’s optimum working pH of 7.4 through the addition of 300 μL of their respective neutralisation buffers. The resulting pH values after extraction were confirmed with a standard glass pH probe and the data is presented in Table 5. For illustrative purposes, a flowchart of this process is depicted in the Supplementary Fig. S.2.

**Table 5:** Dichotomous pH extraction procedure to extract purified versions of either of the pyridine nucleotides, that is, NAD^+^ or NADH in consideration of Lowry *et al*.^62^ and Lilius *et al*.^25^ in three fold repetition.

Extraction Buffer added (mL)	NAD^+^ extraction (pH)	NADH extraction (pH)
0 mL (plasma pH)	7.84 ± 0.05	7.89 ± 0.03
1 mL	4.96 ± 0.06	10.29 ± 0.19
2 mL	3.87 ± 0.05	11.48 ± 0.05
3 mL	2.10 ± 0.03	12.22 ± 0.03
4 mL	1.47 ± 0.02	12.58 ± 0.05
5 mL	1.24 ± 0.01	12.67 ± 0.02
6 mL	1.11 ± 0.01	12.81 ± 0.02

### Pipetting and plate reader settings

Before beginning the assaying phase of the method, the MM was prepared on ice without MTT, PMS or ADH, in order to prevent auto-oxidation of PMS or MTT and denaturation of ADH. Prior to pipetting the samples into the plate, the neutralised samples were centrifuged at 16.000 g for 10 min at 4 °C and the supernatant was successively pipetted into the wells. Standard dilutions (S1 to S6) were supplemented with 50 μL of r-SBFA that had been extracted and neutralised in the same manner as the samples, to provide the standard matrix similar to human plasma. Analogously, 50 μL of DEPC water was added to the samples to be measured in order to simulate the DEPC used in the *β*-NAD standard dilutions. Subsequently, ADH, MTT and PMS were added to the MM, 150 μL of which was pipetted into every well, resuspending twice. Afterwards the plate was stored at room temperature in the dark for 5 min prior to assaying. The absorbance of the samples was then measured at 565 nm in the microplate reader Infinite® 200 PRO (Tecan, Switzerland) in a temperature of 25 °C to prevent significant build-up of bubbles, which occurred at 37 °C. Moreover, the Infinite 200 PRO further served to assess fluorescence at 590 nm and perform autofluorescence scans. All measurements were conducted in duplicates and averaged.

### Enzyme kinetics

Since NAD^+^ is the coenzyme in the specific reaction between ADH and ethanol, one can consider the concentration of NAD^+^ to be rate limiting as the ethanol concentration was chosen well beyond the point of saturation in the MM. NAD^+^ can therefore be regarded as the new substrate and, consequentially, be used in a graphical method to obtain the Michaelis Menten constant, *K*_*m*_, and the maximum reaction velocity, *v*_*max*_, from the double reciprocal Lineweaver-Burke plot described in Lineweaver *et al*.^35^. Here, the y-axis is given by *v*^−1^, and *v*_0_ represents the initial reaction velocity and the x-axis is represented by [*NAD*^+^]^−1^, the reciprocal of the analyte concentration. The double reciprocal Lineweaver-Burk plot facilitated the graphical evaluation of the Michaelis Menten constant, *K*_*m*_, given by the negative reciprocal of the x-axis intercept, in addition to the maximum reaction velocity, *v*_*max*_, given by the reciprocal of the y-axis intercept.

### Regression analysis

As illustrated shortly, the use of an ordinary regression line not through the origin (nTTO, *v*_*R*_ = *mx* + *v*_*b*_), as opposed to a regression line through the origin (TTO, *v*_*R*_ = *mx* + 0), was subject to an increased baseline noise and higher eNAD^+^ estimation bias, introduced by the varying albumin concentrations in the standard matrix. Thus, the TTO method allowed for an albumin independent quantification of eNAD^+^. The relative reaction velocity, *v*_*R*_, is made up of the y-axis intersection,*v*_*b*_, and the slope of the absorbance curve, *m*.

### Reliability, reproducibility and linearity

For the sake of evaluating the repeatability and robustness of the assay, a quantification of eNAD^+^ in healthy human heparinised plasma was conducted with eight independent measurements, featuring the thorough and individual conductance of the complete and previously described experimental protocol, including eight independent standard dilutions, as well as eight individually collected human heparinised plasma samples from a singular subject. Subsequently, these data were utilised to obtain the interval were the enzyme kinetics of the assay reaction occurred in a linear fashion, as well as to confirm the linear relationship between the standard *β*-NAD^+^ sequential dilutions and their respective relative reaction velocities.

### NAD^+^ storage stability

Being of considerable controversy, the stability of eNAD^+^ in frozen plasma suffers from a definitive lack of data in the literature. However, upon the conductance of studies involving human samples, such knowledge is not only of paramount ethical importance but also crucial for the validity of designed experiments, in particular for the algorithm used to store such samples. Therefore healthy human heparinised plasma samples were prepared and stored at −80 °C as described above, before being assayed for eNAD^+^. The baseline measurement consisted of an immediate thawing and assaying of the first sample, whilst additional measurements were performed up to three months.

### Evaluation of a fluorimetric alternative

In consideration of Rhodes *et al*.^20^, we explored the ability of a fluorimetric method to sense eNAD^+^ in human heparinised plasma^20^. For this purpose, we replaced MTT in the MM of the colorimetric method with resazurin, which, upon being reduced, forms the highly red fluorescing resorufin. Moreover, we paid close attention to conserve the number of moles of resazurin compared to MTT participating in the reaction as well as the volume of all suspensions that are part of the MM.

### Statistics

Statistical analysis was conducted in Graphpad’s Prism 7 (GraphPad Software, La Jolla, CA, USA). Concerning the evaluation of the eNAD^+^ storage stability, the data were analysed using a two-tailed, paired t-test with confidence limits of CL = 99%, as well as a two-way ANOVA (without repeated measures) adjusted with Tukey’s multiple comparisons test featuring CL = 99%. The statistical evaluation of the assay’s dependence upon the albumin concentration of the standard matrix was conducted using a two-tailed, unpaired t-test with the confidence limits of CL = 99%. With regards to the reproducibility of the aforementioned method, significance was assessed between two neighbouring standard signals using two different methods, namely a simple, two-tailed, unpaired t-test with the confidence limits of CL = 99%, as well as using the more stringent conditions of a two-way ANOVA (without repeated measures) adjusted with Tukey’s multiple comparisons test with CL = 99%. Regression analysis of the calibration working curve was performed with the gold standard of an ordinary least squares fit, yielding a Pearson Correlation Coefficient, *ρ*, as well as a coefficient of determination, *R*^2^, with CL = 99%. Overall, an alpha value of *p* < 0.01 was applied.

## Electronic supplementary material

Supplementary Information
