# Supplementary material for: The nanomolar sensing of nicotinamide adenine dinucleotide in human plasma using a cycling assay in albumin modified simulated body fluids
Source: Sci Rep. 2018 Oct 31;8:16110. doi: 10.1038/s41598-018-34350-6 (PMC6208386; doi:10.1038/s41598-018-34350-6)
Supplement: Supplementary file 1 — Supplementary Information [file 41598_2018_34350_MOESM1_ESM.pdf]

The nanomolar sensing of nicotinamide adenine dinucleotide in human plasma using a cycling assay in albumin modified simulated body fluids

Philipp Brunnbauer[1,+], Annekatrin Leder[1,+], Can Kamalı[1], Kaan Kamalı[1], Eriselda Keshi[1], Katrin Splith[1], Simon Wabitsch[1], Philipp Haber[1], Georgi Atanasov[1], Linda Feldbrügge[1,2], Igor M. Sauer[1], Johann Pratschke[1], Moritz Schmelzle[1], and Felix Krenzien[1,2,*]

[1]Experimental Surgery and Regenerative Medicine, Department of Surgery, Campus Charité Mitte and Campus Virchow-Klinikum, Charité - Universitätsmedizin Berlin, Berlin, 13353, Germany
[2]Berlin Institute of Health (BIH), Berlin, 10178, Germany
[*]felix.krenzien@charite.de
[+]these authors contributed equally to this work

ADDITIONAL FILE 1 — NAD+ ASSAY PROTOCOL

1 **Prepare buffers in DEPC water:**
- (a) Acid Preparation
  - 0.3 N HCl
  - 0.6 N HCl
- (b) Base Preparation
  - 0.3 N KOH
  - 0.6 N KOH
- (c) Extraction Buffers
  - **NAD extraction**: 0.3 N HCl
  - **NADH extraction**: 0.3 N KOH
- (d) Neutralisation Buffers
  - **TEA-HCl**: 0.36 N TEA-HCL, **adjust pH** to 7.4
  - **NAD neutralisation**: 0.36 N TEA-HCl : 0.6 N KOH (ratio 1 : 1)
  - **NADH neutralisation**: 0.36 N TEA-HCl : 0.6 N HCl : DEPC (ratio 46 : 46 : 108)
- (e) Mastermix (MM) Solutions
  - **TEA-Buffer**: 1 : 10 dilution of TEA, **adjust pH** to 7.4
  - **ADH**: 1 : 10 dilution of ADH suspension
  - **PMS**: 10 mg/mL solution
  - **MTT**: 1 mg/mL solution
  - **EtOH**: use 100 % solution **no DEPC**

2 **Prepare NAD+ Standard Matrix**

Prepare r-SBFA in 1000ml DEPC, as in the table below, add HEPES first, then proceed in order, **adjust pH** to 7.4.
- (a) **NaCl**: 5.403 g, **$NaHCO_3$**: 0.740 g, **Na_2HCO_3**: 2.046 g
- (b) **KCl**: 0.225 g, **KH_2PO_4**: 0.138 g
- (c) **$MgCl_2{\cdot}6H_2O$**: 0.311 g
- (d) **HEPES**: 11.928 g
- (e) **$CaCl_2{\cdot}2H_2O$**: 0.388 g
- (f) **Na_2SO_4**: 0.072 g
- (g) **BSA**: 40 g

3 **Standard Matrix Preparation**

Prepare a β-NAD standard dilution series in the following manner.
- (a) Prepare a 1 mg/mL solution of β-NAD in DEPC
- (b) Dilute β-NAD solution 1000 fold (10 µL solution in 9990 µL DEPC)
- (c) Dilute 500 µL in 500 µL DEPC to make standard 1 (**S1**), **repeat according to the table below**:

Standard	β-NAD (nM)	β-NAD (ng/mL)
S1	753.6	50.00
S2	376.8	25.00
S3	188.4	12.50
S4	94.2	6.25
S5	47.1	3.13
S6	23.5	1.56

4 **NAD Assay Procedure**
- (a) Get Heparin Plasma (sample) from −80 °C, thaw at room temperature
- (b) Separate plasma sample for extraction
  - Pipette 300 µL sample in the eppi for NAD extraction
  - Pipette 30 µL sample + 270 µL r-SBFA in the eppi for NADH extraction
- (c) Prepare standard matrix for extraction (Blank)
  - Pipette 300 µL r-SBFA into an eppi
- (d) Extraction
  - **NAD**: add 300 µL 0.3 N **HCl**
  - **NADH**: add 300 µL 0.3 N **KOH**
  - **r-SBFA**: add 300 µL 0.3 N **HCl**
- (e) Incubation
  - Incubate eppis at 60 °C for 10 min in an eppi heater
- (f) Equilibration
  - Equilibrate eppis **on ice** for 10 min,
- (g) Neutralisation
  - NAD: add 300 µL **NAD Neutralisation Buffer**
  - NADH: add 300 µL **NADH Neutralisation Buffer**
  - r-SBF + A: add 300 µL **NAD Neutralisation Buffer**
- (h) Deproteinisation
  - Centrifuge eppis at 16.000 g for 10 min at 4 °C
  - Transfer supernatant into new eppis
- (i) Get a **transparent multiwell plate and prepare wells in duplicates**
  - **NAD:** 50 µL of Sample, **NADH:** 5 µL of Sample and 45 µL of Blank (1 : 10 Dilution)
- (j) Stop the time, add 150 µL MM to wells, **resuspend twice!**
- (k) After 5 min, measure at 565 nm in Tecan for 30 min, analyse results from 5 – 25 min (linear range)

Figure S.1. *SUPPLMENENTARY: NAD+ Assay Protocol.*

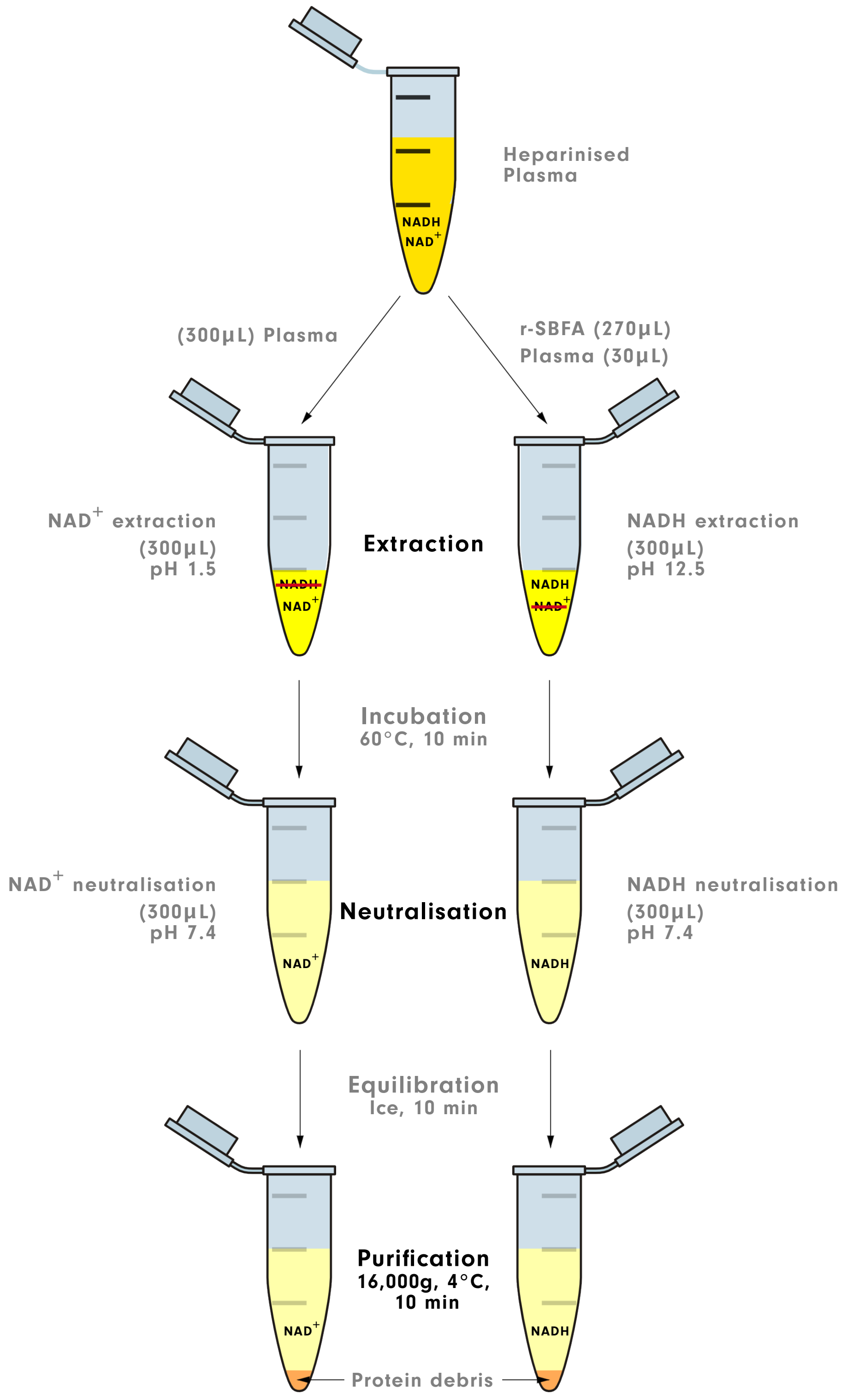

Figure S.2. *NAD^+ and NADH extraction flowchart.* Flowchart of the dichotomous NAD^+ (left path) and NADH extraction (right part) extraction. The NAD^+ and NADH extraction and neutralisation buffers were then prepared in DEPC (Sigma, USA, Catalog No: D5758) water, where the NAD^+ extraction buffer consisted of a 0.3 N HCl solution and the neutralisation buffer comprised equal parts of 0.36 N TEA-HCl (ACROS, USA, Catalog No: 170051000) and 0.6 N KOH. Analogously, 0.3 N KOH was used for the NADH extraction buffer, whilst the neutralisation buffer featured the composition of 23 % 0.36 N TEA-HCl, 23 % 0.6 N HCl and 54 % DEPC water.
